# Meta-analysis of associations between *DLG5* R30Q and P1371Q polymorphisms and susceptibility to inflammatory bowel disease

**DOI:** 10.1038/srep33550

**Published:** 2016-09-16

**Authors:** Yunhai Li, Ping Chen, Jiazheng Sun, Jing Huang, Hongtao Tie, Liangliang Li, Hongzhong Li, Guosheng Ren

**Affiliations:** 1 Chongqing Key Laboratory of Molecular Oncology and Epigenetics, The First Affiliated Hospital of Chongqing Medical University, Chongqing, China; 2 Department of Rheumatology, Daping Hospital, Third Military Medical University, Chongqing, China; 3 Department of Cardiothoracic Surgery, The First Affiliated Hospital of Chongqing Medical University, Chongqing, China

## Abstract

Growing evidence from recent studies has demonstrated an association between inflammatory bowel disease (IBD) susceptibility and two polymorphisms of *DLG5* R30Q (rs1248696) and P1371Q (rs2289310), but the results remain controversial. We conducted a meta-analysis including a total of 22 studies with 10,878 IBD patients and 7917 healthy controls for R30Q and 5277 IBD cases and 4367 controls for P1371Q in order to systematically assess their association with the disease. The results indicated that R30Q was significantly associated with reduced susceptibility to IBD in Europeans by allelic and dominant comparisons, but not in overall population. No significant association was found between R30Q and Crohn’s disease (CD) or ulcerative colitis (UC). P1371Q was associated with increased risk of IBD in Europeans and Americans. On the contrary, a decreased risk of IBD was observed in Asian population for P1371Q. In disease subgroup analysis, we found that P1371Q was also significantly associated with CD, but this relationship was not present for UC. In conclusion, our results strongly suggest that the both polymorphisms of *DLG5* are correlated with IBD susceptibility in an ethnic-specific manner. Additional well-designed studies with large and diverse cohorts are needed to further strengthen our findings.

Inflammatory bowel disease (IBD) is a relapsing and chronic inflammatory disorder mainly affecting the small intestine and colon^1^. Crohn’s disease (CD) and ulcerative colitis (UC) are two major types of IBD. A large number of studies have demonstrated that IBD is caused by multiple reasons including genetic, infectious, environmental and immunoregulatory factors^2^,^3^,^4^. Recently, genome-wide association studies (GWAS) and epidemiological studies have revealed that a considerable number of gene variations, such as in nucleotide oligomerization domain 2 (*NOD2*)^5^,^6^, autophagy related 16-like 1(*ATG16L1*)^7^, signal transducer and activator of transcription 3 *(STAT3*)^8^,^9^, immunity-related GTPase family M (*IRGM*)^10^ and interleukin 23 receptor (*IL23R*)^11^,^12^, are closely related to IBD susceptibility.

Discs large homolog 5 (*DLG5*) gene, a member of the membrane associated guanylate kinase (MAGUK) family, encodes a cell scaffolding protein, which is wildly expressed in human tissues including liver, heart, pancreas, small intestine and colon^13^,^14^. It has been reported that DLG5 participates in various physiological functions such as cell growth, polarity, intracellular signal transduction and the maintenance of epithelial cell integrity^13^,^15^,^16^. In 2004, Stoll and colleagues initially identified several polymorphisms in *DLG5* relating to IBD in a German cohort^16^. In recent years, two of the variants R30Q (rs1248696, G113A), which changes amino acid 30 from arginine (G allele) to glutamine (A allele), and P1371Q (rs2289310, C4136A), which leads to the change of amino acid 1371 from proline (C allele) to glutamine (A allele), have been studied in different populations^17^,^18^,^19^. However, the results remain inconsistent among studies. Since Stoll *et al.* reported that the both variants of *DLG5* were associated with increased risk for IBD, additional studies replicated the association in different populations^16^,^20^,^21^, but the trend was not further verified by other groups with samples from multiple countries^22^,^23^,^24^,^25^,^26^,^27^,^28^,^29^.

Although a meta-analysis performed by Browning *et al.*^30^ attempted to assess the relationship between R30Q and IBD in 2007, they did not observe any significant association. Thus, the conclusion is still doubtful. In addition, there is no meta-analysis investigating the association between P1371Q and IBD up to now. It is necessary to perform a comprehensive study to summarize the overall results of these inconsistent publications. Therefore, we conducted the present meta-analysis to evaluate the potential effects of the two *DLG5* polymorphisms on IBD susceptibility.

## Results

### Study characteristics

**Figure 1:**
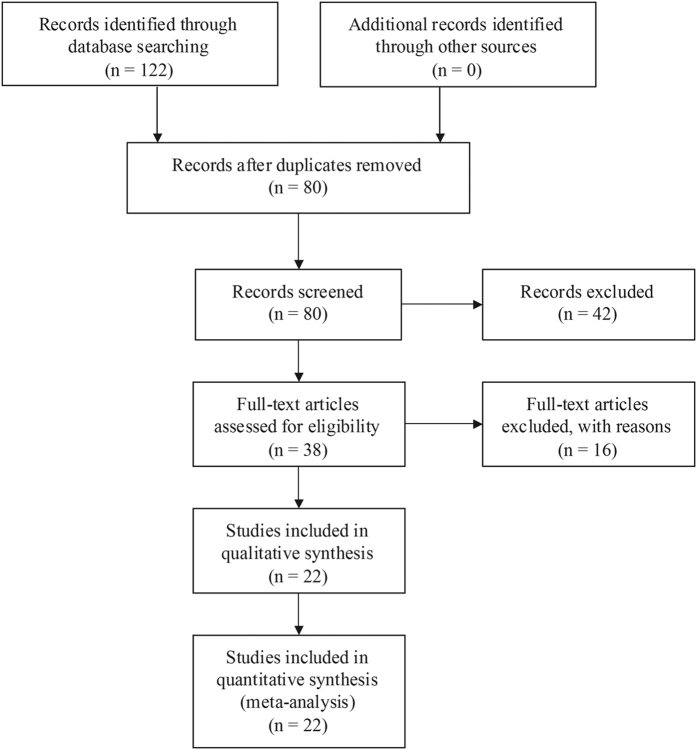
The flow chart of the literature search and study selection process.

Based on our search strategy, 80 articles were retrieved. According to the inclusion and exclusion criteria, 22 articles^16^,^17^,^18^,^19^,^20^,^22^,^23^,^24^,^25^,^26^,^27^,^28^,^29^,^30^,^31^,^32^,^33^,^34^,^35^,^36^,^37^,^38^ with 31 case-control studies were included in this meta-analysis, and 58 studies were excluded for the reason that they were reviews, meeting reports or had no relation to *DLG5* polymorphisms. Specially, for R30Q, two cohorts with detailed allelic and genotype distribution data in the studies of Medici *et al.*^25^ and Pearce *et al.*^29^ were derived from previous publications by Stoll *et al.*^16^ and Daly *et al.*^21^, respectively. Hence, we included the latest and more specific studies of Medici *et al.*^25^ and Pearce *et al.*^29^ in this meta-analysis. The flow chart of the study selection is shown in Fig. 1. Among the 22 eligible studies, 12 investigated the correlation between R30Q and IBD susceptibility, four researched P1371Q and IBD risk, and six studied both R30Q and P1371Q with IBD risk. Finally, a total of 10,878 IBD patients (including 6223 CD and 4551 UC cases) and 7917 controls for R30Q and 5277 IBD patients (including 2745 CD and 1699 UC cases) and 4367 controls for P1371Q were included in the present meta-analysis. The characteristics of the eligible studies are summarized in supplementary Table S1. The allele and genotype distributions of R30Q and P1371Q are listed in supplementary Tables S2 and S3, respectively.

### R30Q polymorphism and IBD susceptibility

The meta-analysis of R30Q was based on a total of 18 articles including 10,878 IBD patients and 7917 controls. Statistical heterogeneity was present in allelic model (*I*^*2*^ = 36%, *P* = 0.050), heterozygote model (*I*^*2*^ = 49%, *P* = 0.009) and dominant model (*I*^*2*^ = 46%, *P* = 0.020). Therefore, random effects model was applied in these analyses. No significant association was identified between R30Q and IBD in overall population (A vs. G: OR = 0.94, 95%CI: 0.86–1.03, *P* = 0.17; GA vs. GG: OR = 0.93, 95%CI: 0.83–1.05, *P* = 0.25; AA vs. GG: OR = 0.88, 95%CI: 0.65–1.18, *P* = 0.38; AA + GA vs. GG: OR = 0.93, 95%CI: 0.83–1.04, *P* = 0.22; AA vs. GA + GG: OR = 0.89, 95%CI: 0.66–1.20, *P* = 0.44). Nevertheless, a trend of reduced risk could be seen (Table 1).

**Figure 2:**
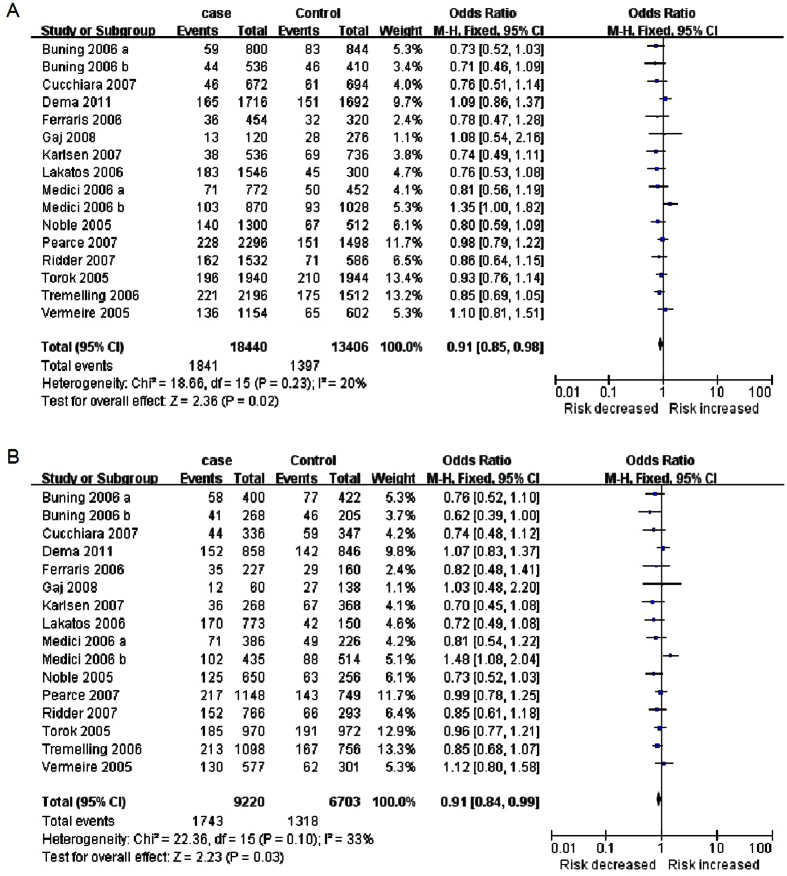
Forest plots of the meta-analysis of R30Q and IBD in European population. (**A**) Allelic model (A vs. G); (**B**) Dominant model (AA + GA vs. GG).

Intriguingly, subgroup analysis based on ethnicity disclosed a significant correlation between R30Q and IBD susceptibility in Europeans in allelic model (A vs. G: OR = 0.91, 95%CI: 0.85–0.98, *P* = 0.02) and dominant model (AA + GA vs. GG: OR = 0.91, 95%CI: 0.84–0.99, *P* = 0.03) by fixed-effect model because no significant statistical heterogeneity was identified (Table 1 and Fig. 2). In order to detect the robustness of the effects, random effects model was used to further analyze the correlation. The results showed that there was still a significant association between R30Q and IBD in Europeans by random effects model (A vs. G: OR = 0.91, 95%CI: 0.83–0.99, *P* = 0.03; AA + GA vs. GG: OR = 0.90, 95%CI: 0.81–0.99, *P* = 0.04). However, no association was observed for the other three genetic models. As for the American and Australasian populations, no significant correlation was found in either cohort (data not shown).

Subsequently, the relationships between R30Q and two major types of IBD (UC and CD) were analyzed. However, no modifying effect of R30Q on the risk of UC or CD was discovered in any genetic model (Table 1).

### P1371Q polymorphism and IBD susceptibility

Ten studies with eleven cohorts including 4985 IBD participants and 4097 controls were utilized to analyze the correlation between P1371Q polymorphism and the risk of IBD. Random effects model was used in the allelic model, homozygote model, dominant model and recessive model due to the presence of heterogeneity, while fixed-effect model was used in heterozygote model. The results showed that P1371Q was not significantly related to IBD in overall population (Table 2). Subgroup analysis indicated an increased risk of IBD in Americans by allele comparison (A vs. C: OR = 1.48, 95%CI: 1.12–1.95, *P* = 0.01), and in Europeans in homozygote comparison (AA vs. CC: OR = 4.06, 95%CI: 1.15–14.29, *P* = 0.03) and recessive comparison (AA vs. CA + CC: OR = 4.05, 95%CI: 1.15–14.26, *P* = 0.03) using fixed-effect model. However, we observed an opposite relationship between P1371Q and IBD in Asian cohort (CA vs. CC: OR = 0.72, 95%CI: 0.54–0.96, *P* = 0.02) (Table 2). Considering the limited number of studies and heterogeneity, these results should be treated with caution.

**Figure 3:**
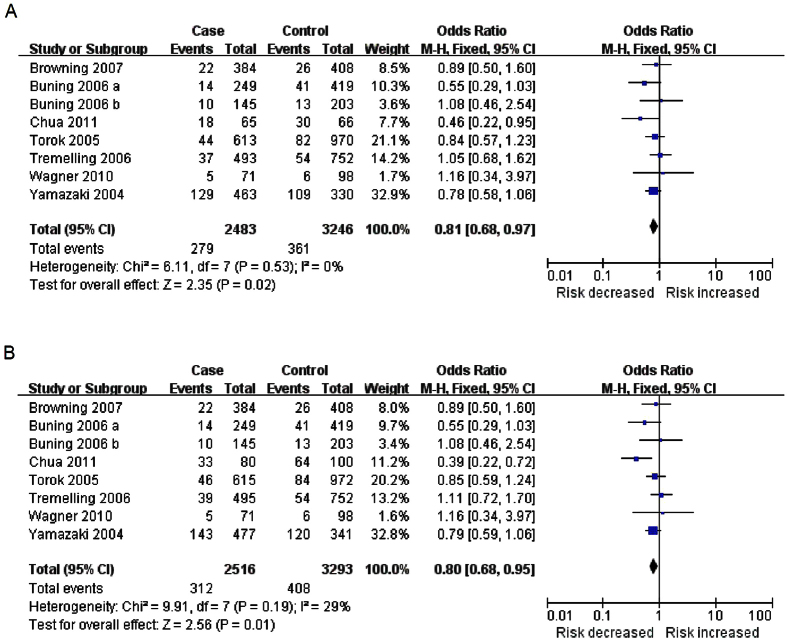
Forest plots of the meta-analysis of P1371Q and CD in overall population. (**A**) Heterozygote model (CA vs. CC); (**B**) Dominant model (AA+CA vs. CC).

Next, the analyses were carried out according to disease type. There were nine studies with 2745 patients and 4097 controls involved in the CD meta-analysis. Interestingly, we detected a significant association between P1371Q and CD in overall population (heterozygote model CA vs. CC: OR = 0.81, 95%CI: 0.68–0.97, *P* = 0.02; dominant model AA + CA vs. CC: OR = 0.80, 95%CI: 0.68–0.95, *P* = 0.01) by fixed-effect model (Table 2 and Fig. 3). Likewise, the reliability of these results was demonstrated by random effects model (CA vs. CC: OR = 0.81, 95%CI: 0.68–0.97, *P* = 0.02; AA + CA vs. CC: OR = 0.80, 95%CI: 0.64–0.99, *P* = 0.04). In addition, a significant statistical association between P1371Q and CD in Asian was identified by heterozygote comparison (CA vs. CC: OR = 0.72, 95%CI: 0.54–0.96, *P* = 0.02). However, no significant association was found in Europeans or Americans (Table 2). For the other major type of IBD, the results did not indicate any statistical association between P1371Q and UC in the overall population, or in Europeans, Americans or Asians for any of the genotype comparisons (Table 2).

### Sensitivity analysis and publication bias

In order to examine the influence set by the two studies^23^,^37^ that did not comply with Hardy-Weinberg equilibrium (HWE) on the pooled effects, sensitivity analysis was performed by omitting them in all genetic models. The results showed that deleting the study of Torok *et al.*^23^ did not materially alter the pooled ORs of R30Q (data not shown). However, the significant effects of P1371Q on CD and IBD disappeared in overall and Asian populations by excluding Chua *et al.*^37^ (data not shown). Egger’s regression test was used to evaluate the publication bias. No significant publication bias was identified in overall comparisons (Tables 1 and 2).

## Discussion

The present meta-analysis examined the association between two crucial polymorphisms of *DLG5* and IBD risk. We uncovered a positive relationship between R30Q and IBD susceptibility in the European subset of cohorts in allelic model and dominant model, which indicated that carriers of the A allele have a lower risk of developing IBD. Moreover, the robustness of the results was also verified by random effects model and a sensitivity analysis by omitting the study derived from HWE. However, no significant correlation was identified in the overall population. It is possible that the presence of allelic heterogeneity among different populations decreased the pooled effects. In addition, subgroup analysis based on disease type did not find a significant relationship between R30Q and CD or UC for the possible reason that stratification reduced the sample size, so the statistics power was insufficient to detect a weak association. As for P1371Q, we demonstrated that this polymorphism increased the risk of IBD in Americans and Europeans, but not in the overall population. On the contrary, P1371Q was associated with decreased risk of IBD in Asians. However, this finding should be interpreted with caution due to the limited number of studies and small sample size. Moreover, in the CD subgroup analysis, we uncovered that P1371Q was significantly related to a lower risk of CD in overall population and Asian ethnicity. But P1371Q did not modify UC risk in all cohorts.

A previous meta-analysis including 13 studies was performed to analyze the association between R30Q polymorphism and IBD risk in Caucasians^30^. However, that study failed to find any evidence for an association. Meanwhile, a case-control study carried out by the same authors found that neither R30Q nor P1371Q was associated with CD, UC or IBD in New Zealand Caucasian population. The main reason for this negative result may be due to the relatively small sample size and lack of deep and complete analysis. Another study focused on the association of R30Q with CD via stratifying the Caucasian population by sex from 12 case-control studies^39^. The authors demonstrated that R30Q was marginally associated with decreased risk for CD in women (*P* = 0.049). Nevertheless, R30Q was not significantly related to CD susceptibility in men (*P* = 0.058). In summary, they provided an evidence of a trend toward reduced CD risk in both males and females. This finding is consistent with our subgroup analysis of CD in European cohort, mainly from Caucasian. Compared to the above two publications, our meta-analysis summarized 22 eligible case-control studies with a larger sample size and offered convincing evidence by systematically analyzing the association of IBD susceptibility with two *DLG5* polymorphisms, including not only the most studied variant, R30Q, but also another significant variant, P1371Q.

As a chronic and relapsing inflammatory disease, a growing number of studies have shown that IBD is associated with both genetic and environmental factors^40^,^41^. Recently, many single nucleotide polymorphisms contributing to the susceptibility of IBD have been identified by genome-wide association study (GWAS) including variants in *DLG5*^11^,^42^,^43^. *DLG5* encodes a cell scaffolding proteins that has been found to play an important role in the determination and maintenance of intestinal epithelial cells polarity^44^. Moreover, *DLG5* participates in epithelial cell proliferation, migration and adhesion^45^. The DLG5 protein contains four domain types, including one DUF622 domain, one SH3 domain, one GK domain and four PDZ domains^46^. The protein–protein interaction domains support DLG5 as a multifunctional adapter and scaffolding protein. Based on observations from *in silico* analysis, both the R30Q and P1371Q variants probably influence or change the structure and function of DLG5^16^. Considering the role of DLG5 in maintaining intestinal barrier integrity^47^, *DLG5* mutations might interfere with the epithelial barrier function of the intestine, and therefore be implicated in IBD pathogenesis.

In 2004, Stoll *et al.*^16^ initially reported that four *DLG5* polymorphisms were associated with IBD in a European cohort, and among them, R30Q was strongly related to an increased risk for IBD. Subsequently, several studies observed similar results^21^,^27^,^48^. However, further studies with samples from different countries attempting to replicate the potential association between R30Q and IBD susceptibility have not been generally successful^22^,^23^,^24^,^26^,^28^,^33^,^36^. One earlier meta-analysis performed by Browning *et al.* summarized R30Q data from published studies and attempted to resolve this discrepancy but failed^30^. In 2008, Browning and colleagues carried out another study by gender-stratified analysis of R30Q in a relatively large cohort^39^. Even so, only a weak relationship was found in women. Simultaneously, the association between P1371Q and IBD has been replicated in only two studies^20^,^27^, and the results of many more studies were inconsistent^18^,^26^,^28^,^30^,^37^,^38^. Since there were so many discrepant results, it was necessary to perform a meta-analysis to better understand the correlation between the two polymorphisms of *DLG5* and IBD susceptibility.

There were several strengths of our meta-analysis. First of all, we performed the present study based on large sample sizes including a total number of 10878 IBD patients with 7917 controls on R30Q polymorphism and 5277 IBD patients with 4367 controls on P1371Q polymorphism, which helped to add convincing evidence. Compared with previous meta-analyses, more studies were included. Additionally, subgroup analysis, sensitivity analysis and genotype comparison were also carried out. Furthermore, we systematically investigated the relationship between P1371Q and IBD risk. To the best of our knowledge, this is the first meta-analysis regarding P1371Q and IBD. Moreover, we set and implemented strict inclusion and exclusion criteria to guarantee the quality of the included studies and the reliability of the results. Finally, selection bias was well-controlled via literature search, and no publication bias was identified by Egger’s test.

Nevertheless, the present meta-analysis has several limitations that should be considered. Firstly, potential heterogeneity observed among the studies of both R30Q and P1371Q, which could not be effectively reduced via subgroup and sensitivity analysis, might partially influence the results. Hence, some other details are needed to analyze the sources of the heterogeneity. For instance, sample size, age of patients and genotyping methods might contribute to the heterogeneity. Secondly, there were two studies with genotypes deprived from HWE, of which one was for R30Q and the other for P1371Q. Although the pooled ORs were not significantly influenced by this study for R30Q, the significant association between P1371Q and CD was diminished by omitting the study for P1371Q. Thirdly, although we found that individuals with P1371Q variant have lower risk of suffering IBD in Asian population, the number of eligible studies and patients was limited. Therefore, many more high quality case-control studies are needed to further explore the association between P1371Q and IBD susceptibility in Asian cohorts.

In summary, despite the limitations above, our results suggest that the *DLG5* R30Q polymorphism is associated with a reduced risk of IBD in Europeans, while P1371Q is associated with an increased risk in Europeans and Americans, but could serve as a protective factor in Asian population. Particularly, P1371Q may decrease the risk of CD in overall population. However, caution should be taken for P1371Q because of insufficient data and population heterogeneity, which might have impact on the convincingness of the results. Well-designed case-control studies with larger sample sizes and multiple ethnicities are required to strengthen the results of the current study. More importantly, studies focusing on the biological functions and mechanisms involving the IBD pathogenesis of DLG5 are also urgently needed.

## Materials and Methods

### Literature search

Two independent investigators conducted a literature search for relevant studies evaluating the relationship between *DLG5* polymorphisms (R30Q and P1371Q) and IBD risk in the Pubmed and Web of Science up to March 14, 2016. The following search terms were used: (inflammatory bowel disease OR IBD OR ulcerative colitis OR UC OR Crohn’s disease OR CD) and (genetic polymorphism OR polymorphism OR variant) and (DLG5 OR discs large homolog 5), without any limitation. Manual search was also performed to obtain additional publications through the reference lists of retrieved studies and reviews.

### Inclusion and exclusion criteria

Studies meeting the following criteria were included: (1) cases-control study; (2) evaluation the association between *DLG5* R30Q or P1371Q polymorphism and IBD risk; (3) provided detail genotype or allelic distribution; (4) contained sufficient data to calculate odds ratios (ORs) and 95% confidence intervals (CIs). Exclusion criteria: (1) reviews, meeting articles, laboratory studies or duplication of previous publications; (2) studies without detail genotype data; (3) researches did not focus on *DLG5* R30Q or P1371Q polymorphism; (4) family-based studies. When there were several articles about a study or the same cohort included in two or more publications were identified, only the latest or complete study was selected.

### Data extraction

The following information was independently extracted by two authors from eligible studies: name of the first author, year of publication, country and ethnicity of the study population, genotyping method, number of cases and controls, allele and genotype frequency in cases and controls. Any disagreement was resolved by discussing with a third author to reach a consensus.

### Statistical analysis

The present meta-analysis was conducted according to the PRISMA checklists and following the guideline^49^. ORs and 95%CI were calculated to measure the strength of association between *DLG5* R30Q and P1371Q variants and IBD risk. Meta-analyses were performed by allelic model (R30Q: A vs. G, P1371Q: A vs. C), heterozygote model (R30Q: GA vs. GG, P1371Q: CA vs. CC), homozygote model (R30Q: AA vs. GG, P1371Q: AA vs. CC), dominant model (R30Q: GA + AA vs. GG, P1371Q: CA + AA vs. CC), recessive model (R30Q: AA vs. GC + GG, P1371Q: AA vs. CA + CC). Cochran’s Q statistic and *I*^2^ test were performed to evaluate the heterogeneity among the eligible studies. Statistical heterogeneity was defined as *P* < 0.1 or *I*^*2*^ > 50%. Random effects models or fixed-effect model was used to pool the effect size depending on the existence of statistical heterogeneity or not, respectively. Chi-square test was applied to evaluate the Hardy-Weinberg equilibrium (HWE) in control group. Publication bias was detected by Egger’s test. All the *P* value were two-sided and *P* < 0.05 was considered statistically significant. All statistical analyses were performed by Review Manager software version 5.2 and Stata software version 12.0 (StataCorp, College Station, TX, USA).

## Additional Information

**How to cite this article**: Li, Y. *et al.* Meta-analysis of associations between *DLG5* R30Q and P1371Q polymorphisms and susceptibility to inflammatory bowel disease. *Sci. Rep.* **6**, 33550; doi: 10.1038/srep33550 (2016).

## Supplementary Material

Supplementary Information

## Figures and Tables

**Table 1:** Summary of the association between R30Q and inflammatory bowel disease.

Disease	Comparison	Ethnicity	No. of study	Test of association			Model	Test of heterogeneity		Egger’s test (P-value)
				OR	95% CI	*P-*value		*P-*value	*I*^*2*^	
CD	A vs. G	Overall	20	0.93	0.82–1.05	0.24	R	0.008	49%	0.501
	(Allelic model)	European	16	0.88	0.78–1.00	0.05	R	0.050	40%	0.131
	GA vs. GG	Overall	19	0.93	0.81–1.07	0.32	R	0.008	49%	0.418
	(Heterozygote model)	European	16	0.89	0.77–1.02	0.09	R	0.040	42%	0.094
	AA vs. GG	Overall	19	1.00	0.72–1.39	1.00	F	0.650	0%	0.276
	(Homozygote model)	European	16	0.91	0.64–1.28	0.57	F	0.840	0%	0.234
	AA + GA vs. GG	Overall	19	0.94	0.82–1.07	0.34	R	0.010	47%	0.443
	(Dominant model)	European	16	0.89	0.79–1.01	0.07	R	0.080	36%	0.103
	AA vs. GA + GG	Overall	19	1.02	0.73–1.42	0.92	F	0.650	0%	0.276
	(Recessive model)	European	16	0.92	0.66–1.31	0.66	F	0.820	0%	0.234
UC	A vs. G	Overall	18	0.95	0.87–1.04	0.28	F	0.380	6%	0.628
	(Allelic model)	European	15	0.93	0.84–1.02	0.11	F	0.580	0%	0.699
	GA vs. GG	Overall	17	0.96	0.87–1.06	0.45	F	0.160	26%	0.841
	(Heterozygote model)	European	15	0.94	0.85–1.05	0.28	F	0.320	12%	0.555
	AA vs. GG	Overall	17	0.82	0.56–1.21	0.21	F	0.750	0%	0.883
	(Homozygote model)	European	15	0.76	0.51–1.14	0.19	F	0.820	0%	0.885
	AA + GA vs. GG	Overall	17	0.95	0.87–1.05	0.34	F	0.220	20%	0.820
	(Dominant model)	European	14	0.93	0.84–1.03	0.28	F	0.440	1%	0.568
	AA vs. GA + GG	Overall	17	0.83	0.57–1.22	0.35	F	0.740	0%	0.883
	(Recessive model)	European	15	0.77	0.52–1.15	0.21	F	0.790	0%	0.885
IBD	A vs. G	Overall	20	0.94	0.86–1.03	0.17	R	0.050	36%	0.405
	(Allelic model)	European	16	**0.91**	**0.85**–**0.98**	**0.02**	F	0.230	20%	0.113
	GA vs. GG	Overall	19	0.93	0.83–1.05	0.25	R	0.009	49%	0.671
	(Heterozygote model)	European	16	0.90	0.80–1.01	0.06	R	0.050	40%	0.144
	AA vs. GG	Overall	19	0.88	0.65–1.18	0.38	F	0.550	0%	0.357
	(Homozygote model)	European	16	0.80	0.59–1.09	0.16	F	0.740	0%	0.321
	AA + GA vs. GG	Overall	19	0.93	0.83–1.04	0.22	R	0.020	46%	0.679
	(Dominant model)	European	16	**0.91**	**0.84**–**0.99**	**0.03**	F	0.100	33%	0.154
	AA vs. GA + GG	Overall	19	0.89	0.66–1.20	0.44	F	0.530	0%	0.357
	(Recessive model)	European	16	0.82	0.60–1.11	0.20	F	0.710	0%	0.321

CD: Crohn’s disease; UC: ulcerative colitis; IBD: inflammatory bowel disease; vs.: versus; R: random effects model; F: fixed effect model.

**Table 2:** Summary of the association between P1371Q and inflammatory bowel disease.

Disease	Comparison	Ethnicity	No. of study	Test of association			Model	Test of heterogeneity		Egger’s test (*P*-value)
				OR	95% CI	*P-*value		*P-*value	*I*^*2*^	
CD	A vs. C	Overall	9	0.89	0.68–1.18	0.43	R	0.002	67%	0.899
	(Allelic model)	European	4	0.91	0.72–1.25	0.42	F	0.280	22%	0.761
		American	2	1.30	0.67–2.53	0.44	R	0.060	72%	NA
		Asian	2	0.62	0.34–1.14	0.13	R	0.020	83%	NA
	CA vs. CC	Overall	8	**0.81**	**0.68**–**0.97**	**0.02**	F	0.530	0%	0.898
	(Heterozygote model)	European	4	0.86	0.67–1.10	0.22	F	0.380	2%	0.843
		Asian	2	**0.72**	**0.54**–**0.96**	**0.02**	F	0.190	43%	NA
	AA vs. CC	Overall	8	0.77	0.31–1.96	0.59	R	0.090	54%	0.490
	(Homozygote model)	European	4	2.79	0.59–13.14	0.19	F	0.380	0%	NA
		Asian	2	0.53	0.22–1.29	0.16	R	0.100	62%	NA
	AA + CA vs. CC	Overall	8	**0.80**	**0.68**–**0.95**	**0.01**	F	0.190	29%	0.975
	(Dominant model)	European	4	0.88	0.69–1.12	0.30	F	0.320	14%	0.836
		Asian	2	0.59	0.30–1.15	0.12	R	0.040	75%	NA
	AA vs. CA + CC	Overall	8	0.71	0.44–1.16	0.17	F	0.170	40%	0.490
	(Recessive model)	European	4	2.81	0.60–13.26	0.19	F	0.390	0%	NA
		Asian	2	0.60	0.36–1.01	0.05	F	0.190	41%	NA
UC	A vs. C	Overall	6	1.19	0.97–1.46	0.09	F	0.850	0%	0.737
	(Allelic model)	European	4	1.15	0.89–1.47	0.28	F	0.730	0%	0.800
		American	2	1.31	0.91–1.88	0.15	F	0.560	0%	NA
	CA vs. CC	Overall	5	1.11	0.88–1.40	0.38	F	0.930	0%	0.240
	(Heterozygote model)	European	4	1.10	0.85–1.42	0.49	F	0.840	0%	0.364
	AA + CA vs. CC	Overall	5	1.13	0.90–1.43	0.29	F	0.900	0%	0.278
	(Dominant model)	European	4	1.12	0.87–1.45	0.37	F	0.800	0%	0.406
IBD	A vs. C	Overall	11	1.05	0.83–1.34	0.67	R	0.000	69%	0.398
	(Allelic model)	European	5	1.08	0.90–1.29	0.43	F	0.240	27%	0.670
		American	3	**1.48**	**1.12**–**1.95**	**0.01**	F	0.160	45%	0.806
		Asian	2	0.62	0.34–1.14	0.13	R	0.020	83%	NA
	CA vs. CC	Overall	10	0.95	0.82–1.10	0.50	F	0.120	36%	0.399
	(Heterozygote model)	European	5	0.99	0.82–1.20	0.94	F	0.590	0%	0.540
		American	2	1.47	0.67–3.25	0.34	R	0.080	68%	NA
		Asian	2	**0.72**	**0.54**–**0.96**	**0.02**	F	0.190	43%	NA
	AA vs. CC	Overall	10	1.13	0.40–3.17	0.82	R	0.030	63%	0.240
	(Homozygote model)	European	5	**4.06**	**1.15**–**14.29**	**0.03**	F	0.670	0%	0.503
		Asian	2	0.53	0.22–1.29	0.16	R	0.100	62%	NA
	AA + CA vs. CC	Overall	10	0.98	0.77–1.23	0.84	R	0.020	55%	0.364
	(Dominant model)	European	5	1.04	0.86–1.25	0.72	F	0.390	2%	0.560
		American	2	1.47	0.67–3.25	0.34	R	0.080	68%	NA
		Asian	2	0.59	0.30–1.15	0.12	R	0.040	75%	NA
	AA vs. CA + CC	Overall	10	1.14	0.46–2.81	0.77	R	0.070	55%	0.240
	(Recessive model)	European	5	**4.05**	**1.15**–**14.26**	**0.03**	F	0.680	0%	0.503
		Asian	2	0.60	0.36–1.01	0.05	F	0.190	41%	NA

CD: Crohn’s disease; UC: ulcerative colitis; IBD: inflammatory bowel disease; vs.: versus; R: random effects model; F: fixed effect model; NA: not available.
