# Supplementary material for: Meta-analysis of associations between DLG5 R30Q and P1371Q polymorphisms and susceptibility to inflammatory bowel disease
Source: Sci Rep. 2016 Sep 16;6:33550. doi: 10.1038/srep33550 (PMC5025715; doi:10.1038/srep33550)
Supplement: Supplementary Information [file srep33550-s1.pdf]

Meta-analysis of associations between *DLG5* R30Q and P1371Q polymorphisms and susceptibility to inflammatory bowel disease

Yunhai Li[1], Ping Chen[2], Jiazheng Sun[1], Jing Huang[1], Hongtao Tie[3], Liangliang Li[2], Hongzhong Li[1*] & Guosheng Ren[1*]

Study	Year	Country	Ethnicity	Diseases	Age	Polymorphism	Genotyping method	N. Case			N. Controls	HWE (*P*-value)
								IBD	CD	UC		
Dema	2011	Spanish	European	IBD	Muti-age	R30Q(rs1248696)	Taqman	858	411	447	846	0.63
Wagner	2010	Australia	Australasian	CD	Child	R30Q(rs1248696)	Sequenom	71	71	NA	97	0.60
Lin	2009	America	American	IBD	Muti-age	R30Q(rs1248696)	PCR-RFLP	213	116	97	170	0.67
Gaj	2008	Poland	European	CD	Adult	R30Q(rs1248696)	Taqman	60	60	NA	138	0.93
Karlsen	2007	Norway	European	IBD	Muti-age	R30Q(rs1248696)	Taqman	268	32	236	368	0.75
Ridder	2007	Netherlands	European	IBD	Muti-age	R30Q(rs1248696)	Taqman	766	440	326	293	0.93
Cucchiara	2007	Italy	European	IBD	Child	R30Q(rs1248696)	Taqman	336	165	171	347	0.90
Browning	2007	New Zealand	Australasian	IBD	Muti-age	R30Q(rs1248696)	Taqman	793	388	405	410	0.51
Pearce	2007	United Kingdom	European	IBD	Muti-age	R30Q(rs1248696)	Pyrosequencing	1148	630	518	749	0.99
Tremelling	2006	United Kingdom	European	IBD	Muti-age	R30Q(rs1248696)	Taqman	1098	494	509	756	0.75
Lakatos	2006	Hungary	European	IBD	Adult	R30Q(rs1248696)	PCR-RFLP	773	639	134	150	0.97
Newman	2006	Cananda	American	IBD	Muti-age	R30Q(rs1248696)	Sequenom	581	402	179	537	R

Medici	2006	Norway	European	IBD	Muti-age	R30Q(rs1248696)	Taqman	386	138	248	226	0.49
Medici	2006	Germany	European	IBD	Muti-age	R30Q(rs1248696)	Taqman	435	293	145	514	0.91
Ferraris	2006	Italy	European	IBD	Child	R30Q(rs1248696)	NanoChip	227	134	93	160	0.47
Buning	2006	Germany	European	IBD	Muti-age	R30Q(rs1248696)	FRET	400	250	150	422	0.57
Buning	2006	Hungary	European	IBD	Muti-age	R30Q(rs1248696)	FRET	268	144	126	205	0.19
Vermeire	2005	Belgium	European	IBD	Muti-age	R30Q(rs1248696)	Sequenom	577	445	118	301	0.95
Torok	2005	Germany	European	IBD	Muti-age	R30Q(rs1248696)	RFLP	970	615	355	972	0.04
Noble	2005	United Kingdom	European	IBD	Adult	R30Q(rs1248696)	Taqman	650	356	294	256	0.98
Lin	2011	America	American	IBD	Muti-age	P1371Q(rs2289310)	PCR-RFLP	212	NA	NA	170	0.92
Chua	2011	Malaysia	Asian	CD	NA	P1371Q(rs2289310)	PCR-RFLP	80	80	NA	100	0.00
Wagner	2010	Australia	Australasian	CD	Child	P1371Q(rs2289310)	Sequenom	71	71	NA	98	0.95
Browning	2007	New Zealand	American	IBD	Muti-age	P1371Q(rs2289310)	Taqman	790	384	406	408	0.80
Tremelling	2006	United Kingdom	European	IBD	Muti-age	P1371Q(rs2289310)	Taqman	1098	495	507	752	0.59
Newman	2006	Cananda	American	IBD	Muti-age	P1371Q(rs2289310)	Sequenom	387	229	158	388	R
Buning	2006	Germany	European	IBD	Muti-age	P1371Q(rs2289310)	FRET	399	249	150	419	0.57
Buning	2006	Hungary	European	IBD	Muti-age	P1371Q(rs2289310)	FRET	268	145	123	203	0.89
Torok	2005	Germany	European	IBD	Muti-age	P1371Q(rs2289310)	RFLP	970	615	355	972	1.00
Yamazaki	2004	Japan	Asian	CD	NA	P1371Q(rs2289310)	Sequencing	477	477	NA	341	0.86
Stoll	2004	Germany	European	IBD	Muti-age	P1371Q(rs2289310)	Taqman	525	NA	NA	516	0.84

Supplementary Table S1. Characteristics of the studies included in this meta-analysis for *DLG5* R30Q (rs1248696) and P1371Q (2289310) polymorphisms. CD: Crohn's disease; UC: ulcerative colitis; IBD: inflammatory bowel disease; PCR: polymerase chain reaction; RFLP: restricted fragment length polymorphisms; RFLP: fluorescent resonance energy transfer; NA: not available; HWE: Hardy-Weinberg equilibrium;

R: reported by original article $P > 0.05$.

Study	Disease	Cases						Control				
		G	A	GG	GA	AA		G	A	GG	GA	AA
Dema, 2011	CD	735	87	332	71	8		1541	151	704	133	9
	UC	816	78	374	68	5		1541	151	704	133	9
	IBD	1551	165	706	139	13		1541	151	704	133	9
Wagner, 2010	CD	128	14	57	14	0		176	18	79	18	0
	IBD	128	14	57	14	0		176	18	79	18	0
Lin, 2009	CD	203	29	87	29	0		318	22	148	22	0
	UC	171	23	74	23	0		318	22	148	22	0
	IBD	374	52	161	52	0		318	22	148	22	0
Gaj, 2008	CD	107	13	48	11	1		248	28	111	26	1
	IBD	107	13	48	11	1		248	28	111	26	1
Karlsen, 2007	CD	61	3	29	3	0		667	69	301	65	2
	UC	437	35	203	31	2		667	69	301	65	2
	IBD	498	38	232	34	2		667	69	301	65	2
Ridder, 2007	CD	784	96	349	86	5		515	71	227	61	5
	UC	586	66	265	56	5		515	71	227	61	5
	IBD	1370	162	614	142	10		515	71	227	61	5
Cucchiara, 2007	CD	312	18	148	16	1		633	61	288	57	2
	UC	314	28	144	26	1		633	61	288	57	2

	IBD	626	46	292	42	2		633	61	288	57	2
Browning, 2007	CD	679	97	299	81	8		738	82	330	78	2
	UC	726	84	326	74	5		738	82	330	78	2
	IBD	1405	181	625	155	13		738	82	330	78	2
Pearce, 2007	CD	1138	122	515	108	7		1347	151	606	135	8
	UC	930	106	416	98	4		1347	151	606	135	8
	IBD	2068	228	931	206	11		1347	151	606	135	8
Tremelling, 2006	CD	885	103	397	91	6		1337	175	589	159	8
	UC	919	99	410	99	0		1337	175	589	159	8
	IBD	1975	221	885	205	8		1337	175	589	159	8
Lakatos, 2006	CD	1134	144	506	122	11		255	45	108	39	3
	UC	229	39	97	35	2		255	45	108	39	3
	IBD	1363	183	603	157	13		255	45	108	39	3
Newman, 2006	CD	741	63	NA	NA	NA		980	94	NA	NA	NA
	UC	326	32	NA	NA	NA		980	94	NA	NA	NA
	IBD	1067	95	NA	NA	NA		980	94	NA	NA	NA
Medici, 2006, a	CD	246	30	108	30	0		402	50	177	48	1
	UC	455	41	207	41	0		402	50	177	48	1
	IBD	701	71	315	71	0		402	50	177	48	1
Medici, 2006, b	CD	518	68	225	68	0		935	93	426	83	5
	UC	253	37	109	35	1		935	93	426	83	5
	IBD	767	103	333	101	1		935	93	426	83	5
Ferraris, 2006	CD	245	23	112	21	1		288	32	131	26	3

	UC	173	13	80	13	0		288	32	131	26	3
	IBD	418	36	192	34	1		288	32	131	26	3
Buning, 2006, a	CD	466	34	217	32	1		761	83	345	71	6
	UC	275	25	125	25	0		761	83	345	71	6
	IBD	741	59	342	57	1		761	83	345	71	6
Buning, 2006, b	CD	266	22	124	18	2		364	46	159	46	0
	UC	226	26	103	20	3		364	46	159	46	0
	IBD	492	44	227	38	3		364	46	159	46	0
Vermeire, 2005	CD	788	102	348	92	5		537	65	239	59	3
	UC	204	32	87	30	1		537	65	239	59	3
	IBD	1018	136	447	124	6		537	65	239	59	3
Noble, 2005	CD	639	73	291	57	8		445	67	193	59	4
	UC	521	67	234	53	7		445	67	193	59	4
	IBD	1160	140	525	110	15		445	67	193	59	4

Supplementary Table S2. Allele and genotype information of R30Q among IBD, CD, UC cases and controls. CD: Crohn's disease; UC: ulcerative colitis; IBD: inflammatory bowel disease; NA: not available.

Study	Disease	Case						Control				
		C	A	CC	CA	AA		C	A	CC	CA	AA
Lin, 2011	IBD	397	27	185	27	0		330	10	160	10	0
Chua, 2011	CD	112	48	47	18	15		102	98	36	30	34
	IBD	112	48	47	18	15		102	98	36	30	34

Wagner,2010	CD	137	5	66	5	0		190	6	92	6	0
	IBD	137	5	66	5	0		190	6	92	6	0
Browning, 2007	CD	746	22	362	22	0		790	26	382	26	0
	UC	782	30	376	30	0		790	26	382	26	0
	IBD	1528	52	738	52	0		790	26	382	26	0
Tremelling, 2006	CD	949	41	456	37	2		1450	54	698	54	0
	UC	979	35	472	35	0		1450	54	698	54	0
	IBD	2113	83	1017	79	2		1450	54	698	54	0
Newman,2006	CD	411	47	NA	NA	NA		729	47	NA	NA	NA
	UC	289	27	NA	NA	NA		729	47	NA	NA	NA
	IBD	700	74	NA	NA	NA		729	47	NA	NA	NA
Buning, 2006, a	CD	484	14	235	14	0		797	41	378	41	0
	UC	284	16	134	16	0		797	41	378	41	0
	IBD	768	30	369	30	0		797	41	378	41	0
Buning, 2006, b	CD	280	10	135	10	0		393	13	190	13	0
	UC	235	11	112	11	0		393	13	190	13	0
	IBD	515	21	247	21	0		393	13	190	13	0
Torok, 2005	CD	1182	48	569	44	2		1858	86	888	82	2
	UC	670	40	318	34	3		1858	86	888	82	2
	IBD	1852	88	887	78	5		1858	86	888	82	2
Yamazaki, 2004	CD	797	157	334	129	14		551	131	221	109	11
	IBD	797	157	334	129	14		551	131	221	109	11
Stoll, 2004	IBD	1006	44	486	34	5		1006	26	490	26	0

Supplementary Table S3. Allele and genotype information of P1371Q among IBD, CD, UC cases and controls. CD: Crohn's disease; UC: ulcerative colitis; IBD: inflammatory bowel disease; NA: not available.
